# Development of a novel integrated isothermal amplification system for detection of bacteria-spiked blood samples

**DOI:** 10.1186/s13568-023-01643-7

**Published:** 2023-11-29

**Authors:** Jin Li, Mei-Yun Shang, Shao-Li Deng, Min Li, Ning Su, Xiao-Dong Ren, Xian-Ge Sun, Wen-Man Li, Yu-Wei Li, Ruo-Xu Li, Qing Huang, Wei-Ping Lu

**Affiliations:** Department of Laboratory Medicine, Daping Hospital, Army Medical University (Third Military Medical University), Chongqing, 400042 P.R. China

**Keywords:** Integrated isothermal amplification system, RPA-LFD assays, Common pathogenic bacteria, Bloodstream Infection, Visual detection

## Abstract

Bloodstream infection (BSI) caused by bacteria is highly pathogenic and lethal, and easily develops whole-body inflammatory state. Immediate identification of disease-causing bacteria can improve patient prognosis. Traditional testing methods are not only time-consuming, but such tests are limited to laboratories. Recombinase polymerase amplification combined with lateral flow dipstick (RPA-LFD) holds great promise for rapid nucleic acid detection, but the uncapping operation after amplification easily contaminates laboratories. Therefore, the establishment of a more effective integrated isothermal amplification system has become an urgent problem to be solved. In this study, we designed and fabricated a hermetically sealed integrated isothermal amplification system. Combining with this system, a set of RPA-LFD assays for detecting *S. aureus*, *K. peneumoniae*, *P. aeruginosa*, and *H. influenza* in BSI were established and evaluated. The whole process could be completed in less than 15 min and the results can be visualized by the naked eye. The developed RPA-LFD assays displayed a good sensitivity, and no cross-reactivity was observed in seven similar bacterial genera. The results obtained with 60 clinical samples indicated that the developed RPA-LFD assays had high specifcity and sensitivity for identifying *S. aureus*, *K. peneumoniae*, *P. aeruginosa*, and *H. influenza* in BSI. In conclusion, our results showed that the developed RPA-LFD assay is an alternative to existing PCR-based methods for detection of *S. aureus*, *K. peneumoniae*, *P. aeruginosa*, and *H. influenza* in BSI in primary hospitals.

**Supplementary Information:**

The online version contains supplementary material available at 10.1186/s13568-023-01643-7.

## Introduction

Bloodstream infection (BSI) caused by bacteria can develop clinical symptoms such as chills, high fever, tachycardia and even shock, which can be a serious threat to people’s health (Allerberger and Kern 2020; Kolesnichenko et al. 2021; Shukla et al. 2021). Studies have shown that applying antibiotics within one hour of monitoring a patient for hypotension is beneficial in improving patient survival, otherwise patient mortality increases progressively with the delay in administration (Kumar et al. 2006). Therefore, rapid and accurate identification of pathogenic bacteria is extremely crucial for subsequent medication guidance. For emergency, traditional culture method for diagnosing bacterial infection is not completely satisfactory due to the time-consuming procedures and high-level laboratories, which is unfavorable to timely and rational medication (Palavecino 2020; Peri et al. 2022). Although real-time polymerase chain reaction (real-time PCR) omits culture procedures and shortens turnaround time, real-time PCR still requires sophisticated thermal cycling instruments and is also difficult to be employed without centralized laboratories (Hawkins and Guest 2017; Singh and Roy-Chowdhuri 2016).

To further simplify operational procedures and improve clinical diagnostic performance, a novel isothermal amplification technique, called recombinase polymerase amplification (RPA), was introduced in this study. It relies on three core enzymes containing DNA polymerase, single strand DNA binding protein and recombinase to aid DNA amplification (Piepenburg et al. 2006). Typically, amplifying nucleic acids can be completed under 37–42℃ condition within 20 min (McQuillan and Wilson 2021; Zhang et al. 2021a; Zheng et al. 2021). Fluorescent-based and lateral flow dipstick (LFD)-based detection has been widely established for various targets among the detection format of RPA amplicons (Behrmann et al. 2020; Shelite et al. 2021; Wang et al. 2022; Xu et al. 2021). Herein, to meet the needs for rapid detection on first aid and emergency treatment, especially for resource-limited settings and poorly equipped laboratories, combining RPA assays and LFD strips (designated as RPA-LFD) is a desirable option.

In this study, *Staphylococcus aureus*, *Pseudomonas aeruginosa*, *Klebsiella pneumonia* and *Haemophilus influenzae* were selected as detection targets after reviewing literatures and the BSI bacteria profile of Daping Hospital (Cui et al. 2022; Lisowska-Łysiak et al. 2021; Martinez and Wolk 2016; Mendes et al. 2018; Stryjewski and Boucher 2009; Wisplinghoff et al. 2004). Therefore, a panel of RPA-LFD assays targeting the above four common pathogenic bacteria was developed. Additionally, we described a customized integrated isothermal amplification system for the RPA-LFD assays to minimize cross-contamination risk from reopening the lid after amplification and to ensure results’ accuracy. We determined the sensitivity and specificity of RPA-LFD assays before using the newly developed integrated isothermal amplification system to retrospectively detect 60 bacteria-spiked blood samples.

## Materials and methods

### Primers and NFO probes of RPA

After a systematic literature search and sequence alignment with DNAMAN software, target gene regions were identified for above four common pathogenic bacteria (Fig. S1). *Nuc*, *CelB*, *Eta*, and *Fuck* gene of *S. aureus*, *K. peneumoniae*, *P. aeruginosa*, and *H. influenzae* were respectively selected as the target genes (Gadsby et al. 2015; Jiang et al. 2020a; Meyler et al. 2012; Salman et al. 2013; Song et al. 2000; Tian et al. 2019). Primer sets and corresponding NFO probes were designed. All primers and probes of Basic RPA and RPA-LFD assays were synthesized and purified by Beijing Genomics Institute (BGI) Biotechnology Corporation using polyacrylamide gel electrophoresis (PAGE) and high-performance liquid chromatography (HPLC) respectively. The oligonucleotide sequences of primers and probes of Basic RPA and RPA-LFD assays were showed in Tables 1 and 2.

**Table 1:** Primers for Basic RPA assay

Target bacteria	Target region	Primer name	Sequence (5’- 3’)
*S. aureus*	Nuc	Sau-F1	AGCAAATGCATCACAAACAGATAACGGCGT
		Sau-F2	GATCCAACAGTATATAGTGCAACTTCAACT
		Sau-F3	AATTACATAAAGAACCTGCGACTTTAATTA
		Sau-R1	CCTTGACGAACTAAAGCTTTGTTTACCATT
		Sau-R2	ATGCACTTGCTTCAGGACCATATTTCTCTA
		Sau-R3	TTCTTTGACCTTTGTCAAACTCGACTTCAA
K. *peneumoniae*	CelB	Kpn-F1	CGAATATTCGTGGCGATAACTCGCAAGG
		Kpn-F2	TTACCGCCATTCTGGTGGCGATAATTTCAAC
		Kpn-F3	TTCAATCCCTGGCCAATGGCTGGGGCCCAT
		Kpn-R1	CGGAGATAATTGTAAACAGCGTCAGGATGG
		Kpn-R2	ATGCTCCCATAACCAACGCTGGTAATGACT
		Kpn-R3	CAAGTGATGTTAGCGGAACCTGGATTAAT
*P. aeruginosa*	Eta	Pae-F1	CGAGAAGCCTTCGAACATCAAGGTGTTCAT
		Pae-F2	CTGAACGCCGGTAACCAGCTCAGCCACATG
		Pae-F3	TCTACACCATCGAGATGGGCGACGAGTTGC
		Pae-R1	ATGGCTGATGGCGAGCGTCGGCTGCATCTC
		Pae-R2	GCGGCTGGGCCTGGGCCATGACCACGCTGA
		Pae-R3	GGCACAACACCTTGCCGCTGGCCCATTCGC
H. *influenzae*	Fuck	Hin-F1	CGTCAATGCTCACTCAACGCTTAACTGGTC
		Hin-F2	CACTACAGATCACACAATGGCGGGAACATCAAT
		Hin-F3	CAATGATGACAAACCTTACTAGCGGTAATTG
		Hin-R1	GAGTATCATGTCCACAAGAAATGACAGGTAC
		Hin-R2	ATTCAGCCCTGCACCAGACCCAAACACAGC
		Hin-R3	CATTAAGATTTCCCAGGTGCCAGAACTTAAC

F-forward primer; R-reverse primer; P-probe; THF-tetrahydrofuran

**Table 2:** Primers and probes for RPA-LFD assays

Target bacteria	Target region	Primer/Probe	Sequence (5’- 3’)
*S. aureus*	Nuc	LFD-Sau-F3	AATTACATAAAGAACCTGCGACTTTAATTA
		LFD-Sau-R2	[biotin] ATGCACTTGCTTCAGGACCATATTTCTCTA
		NFO-Sau	[FAM]GCGATTGATGGTGATACGGTTAAATTAATG[THF]ACAAAGGTCAACCA[C3spacer]
*K.peneumoniae*	CelB	LFD-Kpn-F2	TTACCGCCATTCTGGTGGCGATAATTTCAAC
		LFD-Kpn-R3	[biotin] CAAGTGATGTTAGCGGAACCTGGATTAAT
		NFO-Kpn	[FAM]AATGTCCCTGAATTTATCTCTAAATCGTTC [THF]CTTCATTGATTCCAG[C3spacer]
*P. aeruginosa*	Eta	LFD-Pae-F2	CTGAACGCCGGTAACCAGCTCAGCCACATG
		LFD-Pae-R3	[biotin]GGCACAACACCTTGCCGCTGGCCCATTCGC
		NFO-Pae	[FAM]CGAAGCTGGCGCGCGATGCCACCTTCTTCG [THF]CAGGGCGCACGAGAG[C3spacer]
*H.influenzae*	Fuck	LFD-Hin-F1	CGTCAATGCTCACTCAACGCTTAACTGGTC
		LFD-Hin-R1	[biotin]GAGTATCATGTCCACAAGAAATGACAGGTAC
		NFO-Hin	[5’FAM]AGCATCGCTGGGTTTAAGTAATAACCATTTCCCT[THF]CTATGCGTTATGCAGGT[C3spacer]

F-forward primer; R-reverse primer; P-probe; THF-tetrahydrofuran

### Bacterial strains and DNA preparation

Bacterial DNA for sensitivity and specificity assays were extracted from standard reference strains: *S. aureus* ATCC29213, *K. pneumonia* ATCC700603, *P. aeruginosa* ATCC27853, and *H. influenza* ATCC49247. DNA of all these bacteria was extracted using TIANamp Bacteria DNA Kit (Tiangen Biotech Co., Ltd., Beijing, China) according to the manufacturer’s instructions (Ahmad et al. 2020). All 60 bacteria-spiked blood samples were collected from Daping Hospital. Bacteria-spiked blood samples were firstly lysed using Red Blood Cell Lysis Buffer (Sansure Biotech Inc., Hunan, China) before bacterial DNA was extracted by TIANamp Bacteria DNA Kit. The extracted DNA was stored at -80℃ until next use.

### RPA conditions

A series of Basic RPA assays were carried out to screen out the best primer sets producing the highest analytical sensitivity. The best primers were chosen for subsequent RPA-LFD experiments. Basic RPA and RPA-LFD reactions were both performed in 50 µL volume using DNA Basic kits and LFD kits respectively (Amp-Future Biotech Co., Ltd., Weifang, China) (Sun et al. 2021). Each basic RPA reaction contained 29.4 µL A buffer, 2 µL forward primer (10 µM), 2 µL reverse primer (10 µM), 2 µL sample, 12.1 µL nuclease-free water. Each RPA-LFD reaction included 29.4 µL A buffer, 2 µL forward primer (10 µM), 2 µL reverse primer (10 µM), 0.6 µL NFO probe (10 µM), 5 µL DNA sample, 8.5 µL nuclease-free water. The above mixed reagents were transferred to the reaction tubes containing a dried enzyme pellet provided by the kit, subsequently 2.5 µL B buffer (280 mM) was added to lids. The tubes were closed carefully, vortexed and centrifuged briefly. They were immediately placed in a matched metal heat block and incubated at 38℃ for 10 min. Nuclease-free water was used as the negative control. Finally, the Basic RPA products were purified by phenol-chloroform method (Solarbio, Beijing, China) and analyzed by 1.5% agarose gel electrophoresis. RPA-LFD products were detected by LFD strips (Amp-Future Biotech Co., Ltd., Weifang, China). The amplicons of RPA-LFD were diluted 20-fold in buffer (Milenia Biotec GmbH, Germany). Then LFD strips were placed vertically in tubes containing the diluted RPA-LFD products for 5 min.

### Specificity and sensitivity of RPA-LFD assays

Each RPA-LFD assay’s specificity was verified by detecting seven similar bacterial genera. A series of bacteria for detecting possible cross reactions were shown in Table S1. DNA extracted from reference bacterial strains was used as the positive control. To evaluate established RPA-LFD assays’ sensitivity from bacteria-spiked blood samples, bacterial DNA was 10-fold diluted to prepare samples of final serial concentrations ranging from 6 × 10^5^ CFU/mL to 6 CFU/mL. RPA-LFD reactions were prepared according to above RPA-LFD conditions. The experiment was repeated three times for low concentration samples.

### Real-time PCR assay

Real-time PCR master mix was prepared, and reaction procedures were set according to the instructions of the real-time PCR kit (Promega, USA). The primers and probes of real-time PCR used in the study are shown in Table S2 (Gadsby et al. 2015; Salman et al. 2013). The reaction was performed on CFX96 real-time PCR detection system (Bio-Rad, USA).

### Evaluating bacteria-spiked blood samples for RPA-LFD assays with the integrated isothermal amplification system

**Fig. 1:**
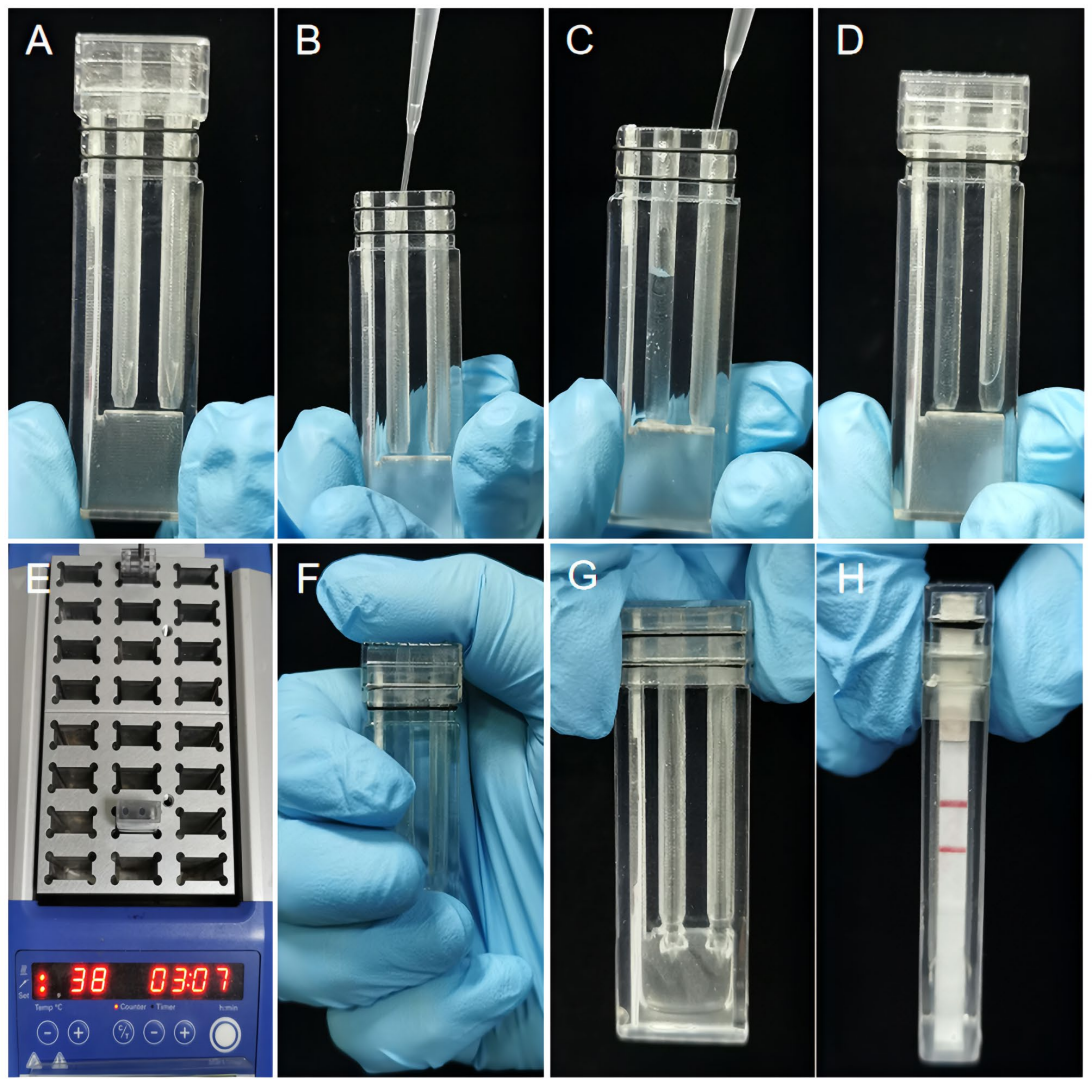
Bacteria-spiked blood samples operation procedures using the integrated isothermal amplification system. **(A)** Placed rubber rings on the detection device and inserted LFD at inspection windows. **(B-C)** RPA reaction mixtures and buffer were added to left channel and right channel of bottom container respectively. **(D)** Inserted top cover into the reaction chamber and sealed with sealing films. **(E)** The assembled detection device was heated in a matched metal heat block at 38 °C for 10 min. **(F-G)** Bottom pad was pierced by top cover. Then, tilting and gently shaking the detection device. **(H)** Observed test results

In order to keep RPA-LFD assays in a closed environment and reduce the problem of cross-contamination, an integrated isothermal amplification system was designed and applied in the RPA-LFD assays. The system integrates RPA assays, LFD strips detecting amplicons, detection devices, and matched metal heat blocks, where the detection device consists of a top cover and a bottom container including a bottom pad, a reaction chamber and an inspection window (Fig. S2). After amplification, results can be interpreted by visualizing the presence or absence of the corresponding bands on the LFD strips with the naked eye. As shown in Figs. 1, 5 µL of DNA template was added to the premixed RPA reaction buffer. Then, the assay device was incubated in metal heat blocks at 38 °C for 10 min. Thereafter, the device was tilted so that the LFD strip located at the inspection window was immersed in the diluted amplicons and the results were interpreted visually based on the presence or absence of bands in the test and control lines. With this integrated isothermal amplification system, the whole process could be completed in less than 15 min, from the time the DNA sample was added into the device to when the reaction result was interpretated. Furthermore, the whole experiment was carried out step by step in a closed space, without aerosol contamination. To perform clinical sample evaluation experiments, 60 clinical samples were collected to construct bacteria-spiked blood samples to verify the feasibility of this new system. The performance of this novel integrated isothermal amplification system was compared with that of mass spectrometry.

**Fig. 2:**
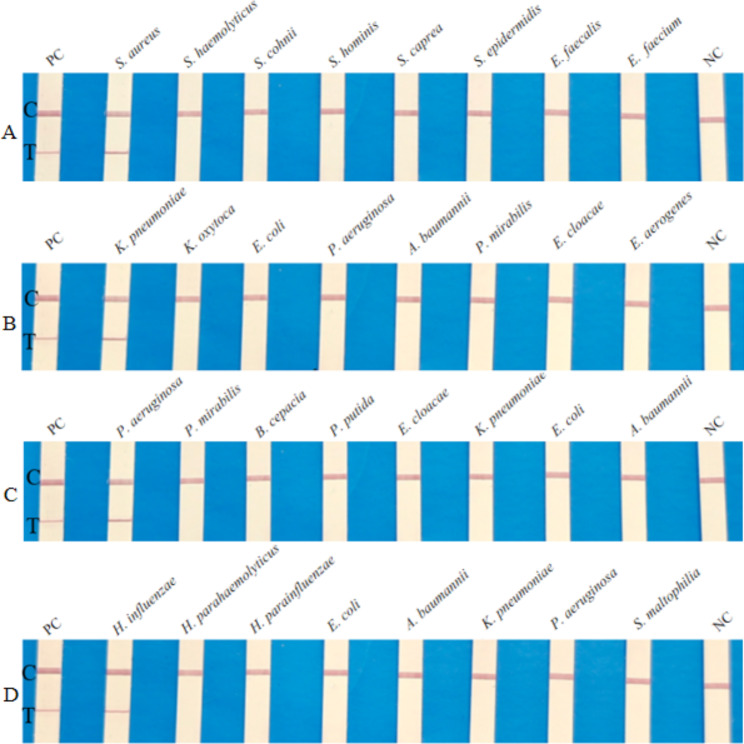
Specificity of RPA-LFD assays. **A, B, C, D** and **E** represent the results of *S. aureus, K. peneumoniae, P. aeruginosa*, and *H. influenzae* RPA-LFD specificity assays, respectively. Results showed that only positive control samples and targeted bacterial samples produced amplification signals, whereas the other pathogen samples and the negative control produced no amplification signals. NC: negative control; C: control line; T: test line

**Fig. 3:**
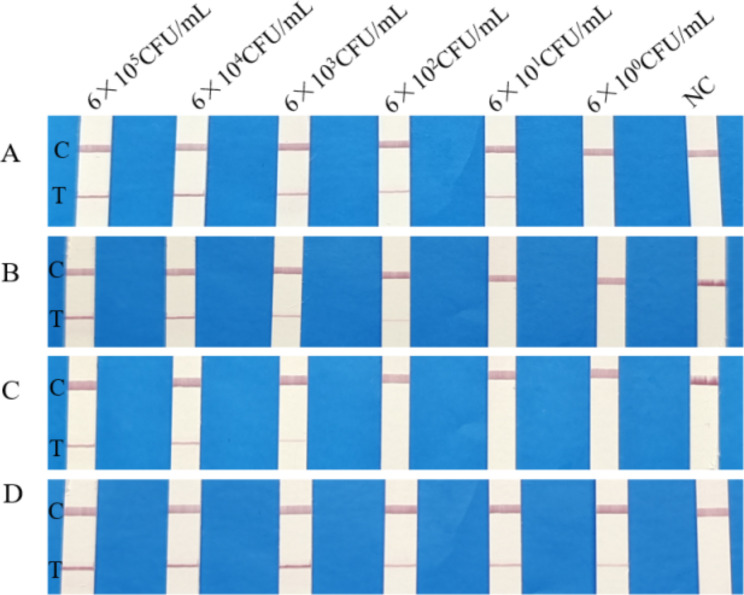
Sensitivity of RPA-LFD assays. **A, B, C, D** and **E** represent the results of *S. aureus, K. peneumoniae, P. aeruginosa*, and *H. influenzae* RPA-LFD sensitivity assays, respectively. Serially diluted DNA concentration of targeted bacteria (6 × 10^5^ CFU/mL, 6 × 10^4^ CFU/mL, 6 × 10^3^ CFU/mL, 6 × 10^2^ CFU/mL, 60 CFU/mL and 6 CFU/mL per reaction) was tested by RPA-LFD assays at 38 °C for 10 min. This experiment was repeated three times for low-concentrated samples (60 CFU/mL-6 CFU/mL). NC: negative control; C: control line; T: test line

**Fig. 4:**
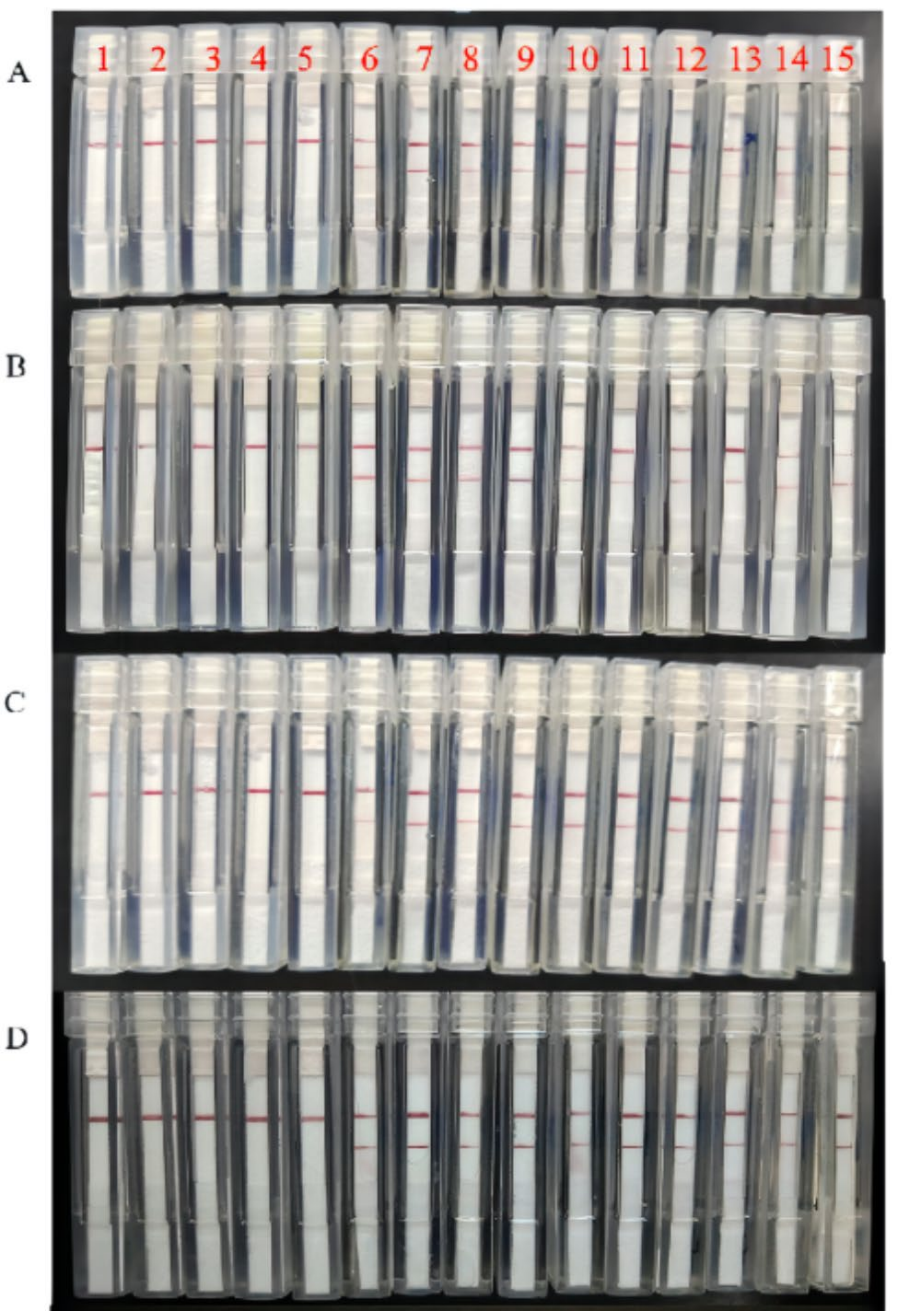
Evaluating bacteria-spiked blood samples for RPA-LFD assays with the integrated isothermal amplification system. **A**: 1, 2, 3, and 4 represent *A. baumannii*, *K. peneumoniae*, *P. aeruginosa*, and *H. influenzae*; 5 represents negative control; 6–15 represent the clinical isolated *S. aureus* samples. **B**: 1, 2, 3, and 4 represent *A. baumannii*, *P. aeruginosa*, *H. influenzae*, and *S. aureus*; 5 represents negative control; 6–15 represent the clinical isolated *K. peneumoniae* samples. **C**: 1, 2, 3, and 4 represent *A. baumannii*, *K. peneumoniae*, *H. influenzae*, and *S. aureus*; 5 represents negative control; 6–15 represent the clinical isolated *P. aeruginosa* samples. **D**: 1, 2, 3, and 4 represent *A. baumannii*, *K. peneumoniae*, *P. aeruginosa*, and *S. aureus*; 5 represents negative control; 6–15 represent the clinical isolated *H. influenzae* samples

## Results

### Primer screening and identification

RPA is a multienzyme-assisted isothermal amplification technique where primers play an important role in unwinding templates, and different primer combinations produce different amplification effects. Therefore, a series of primer screening experiments are essential. Several sets of forward and reverse primer screens were conducted according to the manufacturer’s instructions. As shown in Fig. S3, the best primer sets for *S. aureus, K. peneumoniae*, *P. aeruginosa*, and *H. influenzae* were identified as R2/F3, R3/F2, R3/F2, and R1/F1 based on the recommendations of RPA and the brightness of the electrophoretic bands. The four selected primer sets were used for subsequent RPA-LFD experiments.

### Specificity and sensitivity of RPA-LFD assays

The specificity of RPA-LFD assays for four bacteria species was confirmed by testing seven similar bacterial genera. As can be seen in Fig. 2, only positive control and target bacteria showed test lines, demonstrating that established RPA-LFD reactions have good specificity and no cross reactions occurred. The sensitivity of RPA-LFD assays was determined using a concentration of 6 × 10^5^ CFU/mL to 6 CFU/mL of bacterial DNA extracted from bacteria-spiked blood samples. The results showed that RPA-LFD assays can detect *S. aureus, K. peneumoniae, P. aeruginosa*, and *H. influenzae* with a sensitivity as low as 600 CFU/mL, 60 CFU/mL, 60 CFU/mL and 6 CFU/mL respectively (Fig. 3). By naked eye, the RPA-LFD assays showed sensitivity ranging from 6 × 10^2^ to 6 CFU/mL. The sensitivity of real-time PCR ranged from 6 × 10^3^ to 60 CFU/mL (Fig. S4). Therefore, the sensitivity of RPA-LFD assays was significantly better than that of real-time PCR method.

### Evaluating bacteria-spiked blood samples for RPA-LFD assays with the integrated isothermal amplification system

After the RPA-LFD assays were verified, 10 *S. aureus*-spiked blood samples, 10 *K. peneumoniae*-spiked blood samples, 10 *P. aeruginosa*-spiked blood samples, and 10 *H. influenzae*-spiked blood samples were used for clinical confirmation. In addition, 5 negative controls bacteria-spiked blood samples were made for each RPA-LFD assay to ensure accuracy. From the Fig. 4, it can be seen that the newly RPA-LFD assay had high specifcity and sensitivity for identifying *S. aureus, K. peneumoniae, P. aeruginosa*, and *H. influenzae* in BSI, which may become a powerful tool for rapid and reliable diagnosis of BSI caused by these common pathogenic bacteria in primary hospitals.

## Discussion

Bacterial infections and secondary serious infections from diseases are important causes of death among the elderly, infirm and disabled. Traditional methods and sequencing-based assays for bacterial identification are time-consuming and instrument-intensive, as well as require trained staff, which are not suitable for low-resource areas (Boolchandani et al. 2019; Li et al. 2018; Otašević et al. 2018).

Isothermal amplification techniques, which require much less sophisticated amplification instruments and possess fast reaction kinetics, have been rapidly developed in recent years (Craw and Balachandran 2012; Li and Macdonald 2015; Zhao et al. 2015). Compared with other isothermal amplification assays, the RPA technique used in this study has the shortest reaction time, the simplest experimental design, and the lowest energy consumption (Crannell et al. 2014; Kong et al. 2019). Some studies have shown that experiments can be done using human body temperature (Natoli et al. 2021; Schuler et al. 2015). In just 16 years since its invention in 2006, RPA has reported extensive research on pathogenic microbes and other aspects (Ivanov et al. 2021; Jiang et al. 2020b; Koo et al. 2016; Wang et al. 2021).

In order to develop a set of experimental platforms capable of detecting common bacterial infections for timely treatment of infections, several pairs of primers and their corresponding probes were designed for four bacteria species (*S. aureus, K. peneumoniae, P. aeruginosa*, and *H. influenzae*). The best primer pairs were identified by forward and reverse primer screening experiments using agarose gel electrophoresis and used for subsequent RPA-LFD experiments. The results of specificity assays showed that the four established RPA-LFD assays had no cross reactions with other bacterial species. DNA from bacteria-spiked blood samples was tested for RPA-LFD sensitivity assays. In contrast to previous studies, the matrix of the test samples less involved blood samples, which are an important sample matrix for diagnosing infectious diseases (Chen et al. 2018; Ghosh et al. 2012; Helfrich et al. 2015). Here, to evaluate the practical clinical application of the established RPA-LFD assays, blood samples were used as sample matrix to construct clinical bacteremia samples. Our results showed the sensitivity of established RPA-LFD assays can up to 60 CFU/mL, and even 6 CFU/mL, which was better than that of real-time PCR method. However, the limitation of this study is that the relatively low sensitivity of RPA-LFD in the detection of *S. aureus*. The possible reasons for the low sensitivity may be related to the colony traits of the bacteria themselves, and corresponding nucleic acid extraction protocols should be optimized according to characteristics of different bacteria (Barbaccia et al. 2020; Bogut and Magryś 2021; Chiarelli et al. 2020; Mizukami et al. 2020).

In order to achieve simple and rapid operation, we designed an integrated isothermal amplification system, which consists of RPA assays, LFD strips detecting amplicons, detection devices, and matched metal heat blocks. This system greatly simplifies the detection process of traditional RPA-LFD technique and effectively avoids the contamination caused by amplification products. At the same time, the system developed by us can preliminarily realize the function of point-of-care testing (Chakravorty et al. 2017; Taki et al. 2021; Zhang et al. 2021b). In short, compared with the conventional techniques, such as real-time quantitative PCR, our detection technology has the advantages of simple equipment, rapid operation, and low cost. With the further decline in the price of detection reagents such as recombinant enzymes in the future, the technology will have a lower cost and produce better benefits. Of course, we will continue to improve this work in the future.

In summary, the RPA-LFD assay is time-saving, more effective and sensitive than conventional identification methods, which has the potential to be applied in primary hospitals (Chen et al. 2021; Yin et al. 2017). Moreover, this novel integrated isothermal amplification system will become a powerful tool for the identification of bacteria or other pathogens, especially suitable for use in low-resource settings (Sadaow et al. 2020).

### Electronic supplementary material

Below is the link to the electronic supplementary material.

Supplementary Material 1

Supplementary Material 2

## Data Availability

Data of this study are included in the article and the primary data can be provided from the corresponding author.
