# Supplementary material for: Development of a novel integrated isothermal amplification system for detection of bacteria-spiked blood samples
Source: AMB Express. 2023 Nov 29;13:135. doi: 10.1186/s13568-023-01643-7 (PMC10686969; doi:10.1186/s13568-023-01643-7)
Supplement: Supplementary file 1 — Supplementary Material 1 [file 13568_2023_1643_MOESM1_ESM.docx]

**Title:** Development of a novel integrated isothermal amplification system for detection of bacteria-spiked blood samples

**Authors:** Jin Li^1#^, Mei-Yun Shang^1#^, Shao-Li Deng^1^, Min Li^1^, Ning Su^1^, Xiao-Dong Ren^1^, Xian-Ge Sun^1^, Wen-Man Li^1^, Yu-Wei Li^1^, Ruo-Xu Li^1^, Qing Huang^1*^, Wei-Ping Lu^1*^

***Co-corresponding authors:**

Wei-Ping Lu, M.D. Ph.D., Email: [luweiping19710416@163.com](mailto:luweiping19710416@163.com)

Qing Huang, M.D. Ph.D., Email: [qinghuang@tmm.edu.cn](mailto:qinghuang@tmm.edu.cn)

Tel: +86-23-68746995

Fax: +86-23-68716530

^1^Department of Laboratory Medicine, Daping Hospital, Army Medical University, Chongqing, 400042, P.R. China

^#^These authors contributed equally to this work

# SUPPLEMENTARY MATERIALS INDEX

**Supplementary Figure 1 Relative positions of the amplicon target genes in bacterial specific target genes of bloodstream infection.**

**Supplementary Figure 2 Structure diagram of the detection device.**

**Supplementary Figure 3 Primer screening for basic RPA assays.**

**Supplementary Figure 4 Sensitivity of the real-time PCR assays.**

**Supplementary Table 1 Samples for specificity assays.**

**Supplementary Table 2 Primers and probes of real-time PCR.**


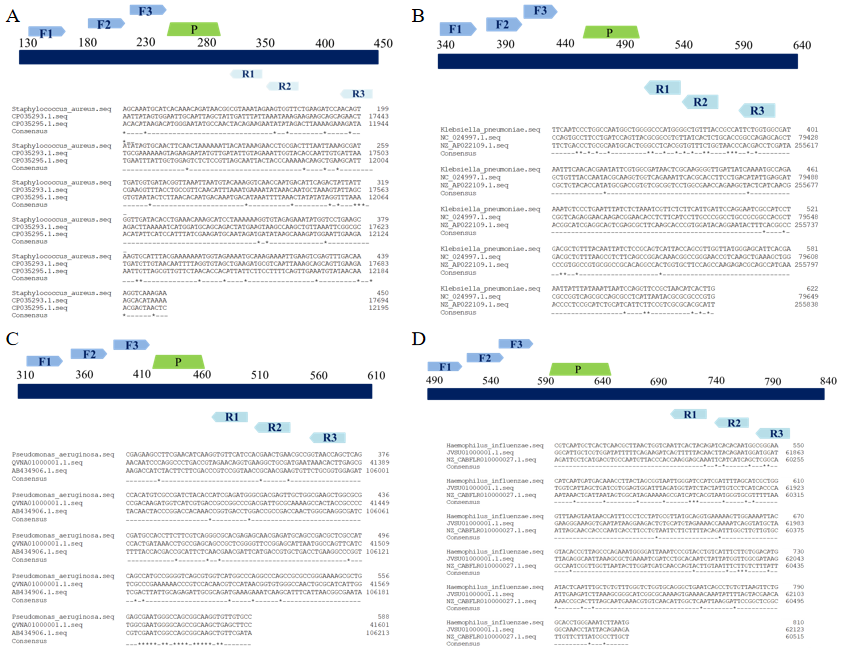


**Supplementary Figure 1. Relative positions of the amplicon target genes in bacterial specific target genes of bloodstream infection**. (**A:** *S. aureus*; **B:** *K. peneumoniae*; **C:** *P. aeruginosa*; **D:** *H. influenzae*).


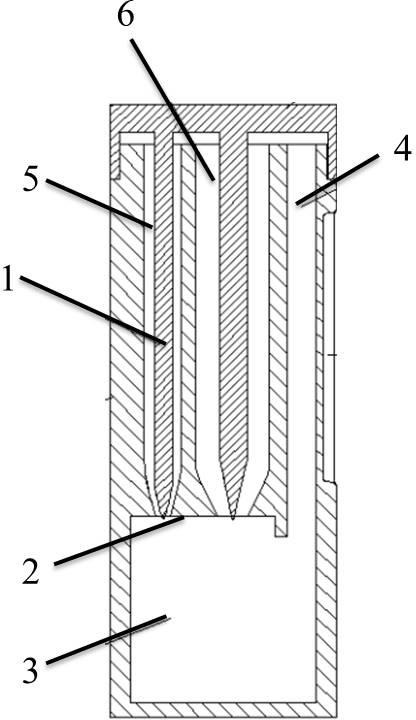


**Supplementary Figure 2. Structure diagram of the detection device.** This device consists of top cover **(1)** and bottom container. Bottom pad **(2)**, reaction chamber **(3)** and inspection window **(4)** constituted the bottom container. Number **5** and **6** represent the left channel and the right channel respectively.


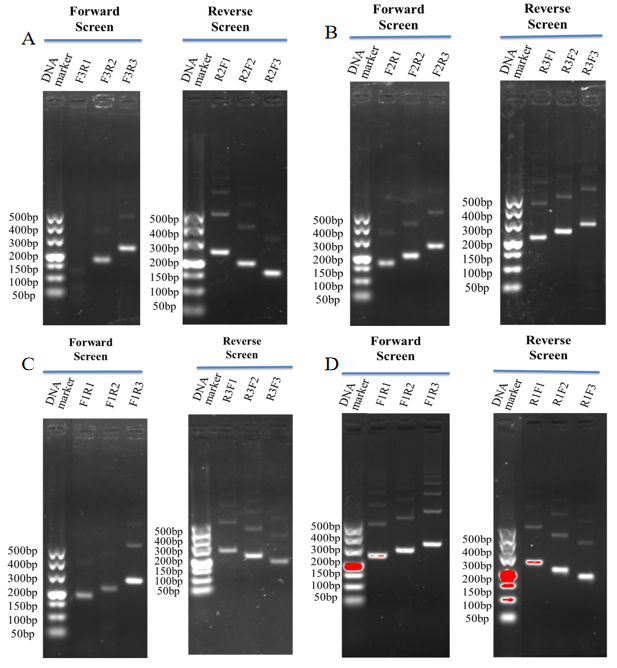


**Supplementary Figure 3. Primer screening for basic RPA**. Several sets of forward and reverse primer screens were conducted according to the manufacturer’s instructions to obtain best amplification efficacy of four bacteria. (**A:** *S. aureus*; **B:** *K. peneumoniae*; **C:** *P. aeruginosa*; **D:** *H. influenzae*).


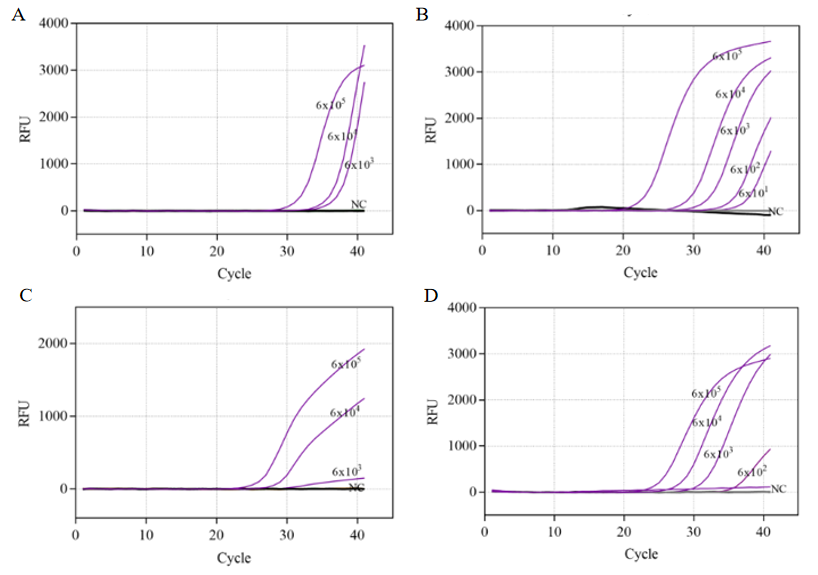


# Supplementary Figure 4. Sensitivity of the real-time PCR assays. A, B, C, D and E represent the results of *S. aureus*, *K. peneumoniae*, *P. aeruginosa*, and *H. influenza* real-time PCR sensitivity assays, respectively. The sensitivity of the real-time PCR assays based on the quantity of genomic DNA of above four bacteria was tested by real-time PCR at 95°C for 2 min, followed by 40 cycles of 95°C for 15 seconds and 60°C for 1 min. NC: negative control.

# Supplementary Table 1 Samples for specificity assays

| **Target bacteria** | **Non-target bacteria** |
| --- | --- |
|  | *Staphylococcus haemolyticus* |
|  | *Staphylococcus cohnii* |
|  | *Staphylococcus hominis* |
| *Staphylococcus aureus* | *Staphylococcus caprea* |
|  | *Staphylococcus epidermidis* |
|  | *Enterococcus faecalis* |
|  | *Enterococcus faecium* |
|  | *Klebsiella oxytoca* |
|  | *Escherichia coli* |
|  | *Acinetobacter baumannii* |
| *Klebsiella peneumoniae* | *Pseudomonas aeruginosa* |
|  | *Proteus mirabilis* |
|  | *Enterobacter cloacae* |
|  | *Enterobacter aerogenes* |
|  | *Proteus mirabilis* |
|  | *Burkholder cepacia* |
|  | *Pseudomonas putida* |
| *Pseudomonas aeruginosa* | *Enterobacter cloacae* |
|  | *Klebsiella pneumoniae* |
|  | *Escherichia coli* |
|  | *Acinetobacter baumannii* |
|  | *Haemophilus parahaemolyticus* |
|  | *Haemophilus parainfluenzae* |
|  | *Escherichia coli* |
| *Haemophilus influenzae* | *Acinetobacter baumannii* |
|  | *Klebsiella pneumoniae* |
|  | *Pseudomonas aeruginosa* |
|  | *Stenotrophomonas maltophilia* |

**Supplementary Table 2 Primers and probes of Real-Time PCR.**

| **Target bacteria** | **Target region** | **Oligonucleotide** | **Origin** |
| --- | --- | --- | --- |
| *S. aureus* | Nuc | Forward:AGCATCCTAAAAAAGGTGTAGAGA  Reverse:CTTCAATTTTMTTTGCATTTTCTACCA  Probe:TEX-TTTTCGTAAATGCACTTGCTTCAGGACCA-BHQ1 | (1) |
| *K. peneumoniae* | CelB | Forward:TTGCCGCCAGCTATCTTCAC  Reverse:GGCAATACGCCAATGACCGT  Probe:FAM-AACCGATGGCTTATGGCGTGCCGCT-BHQ1 | This publishment |
| *P. aeruginosa* | Eta | Forward:ACAACGCCCTCAGCATCAC Reverse:AGTTCAGCGACCAACTGCC  Probe:FAM-TCGAAGGTGGCGTCGAGCCGAACAA-BHQ1 | This publishment |
| *H. influenzae* | Fuck | Forward:ATGGCGGGAACATCAATGA  Reverse:ACGCATAGGAGGGAAATGGTT Probe:FAM-CGGTAATTGGGATCCAT-MGB | (1) |

**Reference**

1. Gadsby NJ, McHugh MP, Russell CD, Mark H, Conway Morris A, Laurenson IF, et al. Development of two real-time multiplex PCR assays for the detection and quantification of eight key bacterial pathogens in lower respiratory tract infections. Clin Microbiol Infect 2015;21(8):788.e1-788.e13. doi: 10.1016/j.cmi.2015.05.004.
