# Supplementary material for: Development of a novel integrated isothermal amplification system for detection of bacteria-spiked blood samples
Source: AMB Express. 2023 Nov 29;13:135. doi: 10.1186/s13568-023-01643-7 (PMC10686969; doi:10.1186/s13568-023-01643-7)
Supplement: Supplementary file 2 — Supplementary Material 2 [file 13568_2023_1643_MOESM2_ESM.pdf]

中国人民解放军陆军特色医学中心伦理委员会
涉及人的生物医学研究伦理审查意见

医研伦审（2021）第223号

研究项目名称	一种快速准确检测病原微生物的便携式恒温扩增一体化检测系统的研制及应用		
项目来源	重庆市科卫联合医学科研项目（2022QNXN034）		
项目研究单位	陆军特色医学中心		
项目负责人	李进	承担科室	检验科
研究时间	2021.11-		
审查方式	快速审查		
审查材料	研究方案		
	免除知情同意申请		

审查意见：

“一种快速准确检测病原微生物的便携式恒温扩增一体化检测系统的研制及应用”送审材料经伦理委员会审查：符合伦理学要求，同意按此方案进行研究。

知情同意书获取方法：■ 适当 □ 不适当

主任委员签名：[illegible]

日期：2021.11.16.

中国人民解放军陆军特色医学中心伦理委员会（盖章）

声明：本伦理委员会的组成和工作程序符合涉及人的生物医学研究审查要求和国家相关法律法规

（注：本批件有效期为一年，逾期未实施的，则自行废止）
